# Physical activity and the development of general mental health problems or attention-deficit hyperactivity disorder (ADHD) symptoms in children and adolescents: A cross-lagged panel analysis of long-term follow-up epidemiological data

**DOI:** 10.3389/fnbeh.2022.933139

**Published:** 2022-09-13

**Authors:** Parisa Ganjeh, York Hagmayer, Thomas Meyer, Ronny Kuhnert, Ulrike Ravens-Sieberer, Nicole von Steinbuechel, Aribert Rothenberger, Andreas Becker

**Affiliations:** ^1^Department of Child and Adolescent Psychiatry and Psychotherapy, University Medical Center Göttingen, Göttingen, Germany; ^2^Department of Cognitive Science and Decision Psychology, Georg-Elias-Müller-Institute for Psychology, University of Göttingen, Göttingen, Germany; ^3^Department of Psychosomatic Medicine and Psychotherapy, University Medical Center Göttingen, Göttingen, Germany; ^4^Unit Mental Health, Department of Epidemiology and Health Monitoring, Robert Koch Institute, Berlin, Germany; ^5^Department of Child and Adolescent Psychiatry, Psychotherapy and Psychosomatics, University Medical Center Hamburg-Eppendorf, Hamburg, Germany; ^6^Institute of Medical Psychology and Medical Sociology, University Medical Center Göttingen, Göttingen, Germany

**Keywords:** physical activity, mental health problems, ADHD, longitudinal relationship, children, adolescents

## Abstract

Studies have shown that physical activity (PA) can provide a helpful, low-risk, and cost-effective intervention for children and adolescents suffering from mental health problems. This longitudinal study aimed to assess whether PA prevents the development of mental health problems, such as attention-deficit/hyperactivity disorder (ADHD) in children and adolescents. Data were analyzed from the German Health Interview and Examination Survey for Children and Adolescents (KiGGS) collected from more than 15.000 children and adolescents at three different time points over a period of more than 10 years. Parents scored the PA of the study participants on three frequency levels according to WHO recommendations, and mental health problems were assessed by means of the Strengths and Difficulties Questionnaire (SDQ). The total problem score (SDQ-Total) and the hyperactivity/inattention symptoms sub-scale (SDQ-H/I) were used in an autoregressive cross-lagged model to examine their relationship with PA. The results showed that PA of boys and girls at preschool age was inversely associated with the occurrence of mental health problems and, in particular, ADHD symptoms about 6 years later. Higher levels of PA were associated with better general mental health and fewer ADHD symptoms at the next time point (Wave 1). These effects were not observed from preadolescence (Wave 1) to adolescence (Wave 2), neither for girls nor for boys. These findings indicate that medium-to-high PA may be a supportive factor for good mental health in children in preschool and elementary school. Future studies will have to show whether PA may be a helpful add-on for interventional programs for improving general mental health and alleviating ADHD symptoms among children and adolescents.

## Introduction

Based on the report of the World Health Organization (WHO), mental health disorders will be the primary cause of disease burden by 2030 (World Health Assembly, 2012). It is estimated that about 10% of children and adolescents have one or more mental health conditions (World Health Organization, 2021). According to the German Robert Koch Institute, about 20% of children and adolescents showed signs of mental health problems between 2003 and 2007 and 17% between 2014 and 2017 (Klipker et al., 2018). These conditions can have adverse implications on social and educational skills, productivity (World Health Organization, 2021), and disabilities (Erskine et al., 2015). These conditions remain stable at best or become more severe later in life (Doering et al., 2019; Khan and Burton, 2021). Epidemiological research is needed to find better ways to improve mental health in children and adolescents.

Physical activity (PA) may be a promising factor within this context (Larun et al., 2006; Brown et al., 2013; Wu et al., 2017; Bélair et al., 2018; Bell et al., 2019; Biddle et al., 2019; Parker et al., 2019; Andermo et al., 2020; Ma et al., 2020; Pascoe et al., 2020; Wegner et al., 2020; Bourke et al., 2021; Heinze et al., 2021; Yang et al., 2021). Systematic reviews and meta-analyses showed positive effects of PA on mental health (Wilson and Barnett, 2020; Brylka et al., 2021; Carter et al., 2021). Even during the COVID-19 pandemic, studies showed that PA can be a protective factor against psychological problems among children and adolescents. In their rapid systematic review, Wolf et al. demonstrated a 12–32% and a 15–34% lower hazard ratio for depression and anxiety, respectively, among those who engaged in regular PA with higher frequency during COVID-19 (Wolf et al., 2021). A number of studies from China and the USA. reported that children and adolescents, who engaged in more PA during the pandemic, reported fewer behavioral problems, mental health problems (both externalizing and internalizing), and negative mood states including tension, depression, anger, fatigue, and confusion (Zhang et al., 2020; Liu et al., 2021; Qin et al., 2021; Tandon et al., 2021).

Whereas temporal or cause-effect relationships between variables cannot be inferred from cross-sectional studies, intervention and longitudinal studies may be appropriate to provide some insight into the likely causality or temporality of the role of PA in preventing mental health problems. Given the above-mentioned findings for a positive effect of PA on mental health, it would be unethical to include a control group with extremely low levels or even without PA for an extended period of time in a randomized controlled trial. Indeed, the design of acceptable control groups in this domain is controversial (Lawlor and Hopker, 2001; Schuch et al., 2016). As an alternative, observational studies with a longitudinal design and an adequate statistical approach (especially cross-lagged models) seem to be more acceptable and still informative.

Several longitudinal studies showed a long-term relationship between PA and psychological constructs, but the results are inconsistent (Wu et al., 2018, 2021; Doré et al., 2019, 2020; Kleppang et al., 2019; Loewen et al., 2019; Gómez-Baya et al., 2020; Hamer et al., 2020; Isaksson et al., 2020; Kandola et al., 2020; van Woudenberg et al., 2020; Bowe et al., 2021). To illustrate some results, Nigg et al. concluded that PA may not be a preventive component for mental health problems, but mental health problems can be a risk factor for lower activity at preadolescent ages (Nigg et al., 2021). The authors created a path panel prediction model using MOMO (Motorik-Modul) data (self-reported data from 686 adolescents aged 11–17 years) of the German KiGGS study and found that PA at Baseline positively predicted prosocial behavior at the next time point exclusively among boys. The inverse result was found for girls. Mental health problems at Baseline negatively predicted PA at the second time point among boys and girls. PA at the third time point was predicted by mental health problems at the second time point only among girls. In another cohort study, the PA in 928 adolescents aged 12**–**13 had a positive association with the emotional sub-scale of the SDQ, but not with the total difficulties score and mental well-being 3 years later (Bell et al., 2019). Moreover, some studies reported no or inverse sequential association between PA and mental health problems (Hinkley et al., 2017; Ahn et al., 2018; Kleppang et al., 2019; Gómez-Baya et al., 2020). These inconsistencies demand more empirical research to give a clearer picture of the relationship between PA and the development of later mental health problems.

Attention-deficit/hyperactivity disorder (ADHD) is one of the most common neurodevelopmental disorders (Thapar et al., 2017). The global prevalence of ADHD is around 5% (Posner et al., 2020), and it has been reported in up to 7.2% among children and adolescents (Thomas et al., 2015). In Germany, the prevalence rate in children and adolescents was 5.3% (2003**–**2006) and 4.4% (2014**–**2017), respectively (Göbel et al., 2018). Age-inappropriate behaviors, including hyperactivity, impulsivity, and inattention, which persist for at least 6 months, are primary ADHD symptoms (American Psychiatric Association, 2016). ADHD has negative effects on social and academic competence, along with a higher risk of mental illness, delinquency (American Psychiatric Association, 2016), and occupational impairment (Erskine et al., 2016). If children with ADHD do not receive effective and timely intervention, at least 60–70% will carry symptoms and psychosocial problems into adulthood (Wilens and Spencer, 2010; Groß et al., 2015; Banaschewski et al., 2017). Given that established treatments for ADHD (i.e., behavior therapy and/or medication) cannot address the whole range of problems, alternative and/or supplementary interventions should be looked for (Rommel et al., 2015; Vysniauske et al., 2020; Welsch et al., 2021).

A growing number of studies provide information that PA may be a promising factor within a treatment program for ADHD symptoms (Halperin et al., 2014; Khalife et al., 2014; Cerrillo-Urbina et al., 2015; Rommel et al., 2015; Ng et al., 2017; Ahn et al., 2018; Wu et al., 2018, 2021; Biddle et al., 2019; Neudecker et al., 2019; Miklós et al., 2020; Vysniauske et al., 2020; Mercurio et al., 2021). Biddle et al. concluded from systematic reviews and meta-analyses that PA leads to better cognitive results and academic achievement (Biddle et al., 2019). Congruent with this conclusion, some studies showed an association between PA and executive and cognitive functions (van der Niet et al., 2015; Xiong et al., 2017; Erickson et al., 2019; Takacs and Kassai, 2019; Xue et al., 2019). Interestingly, in a systematic review and meta-analysis of four non-pharmacological treatments for cognitive problems in ADHD (neurofeedback, cognitive-behavioral therapy, cognitive training, and physical exercises) among 19 selected studies, physical exercise had the highest effect size (Morris d=0.93) among children, adolescents, and adults (Lambez et al., 2020).

A recent study indicated a stronger and more long-lasting improvement in executive functions and behavioral symptoms in children with ADHD when PA was combined with cognitive tasks (Nejati and Derakhshan, 2021). The latest systematic review and meta-analysis also found an improvement in executive functions in 664 children and adolescents with ADHD through exercise intervention (Liang et al., 2021). Significant moderate-to-large effects were reported for inhibitory control and cognitive flexibility. The intensity of exercises and whether the sessions are acute or chronic impacted the results more than the type of exercise. The results of another systematic review showed positive effects of a 1-h long PA and short duration activities on attention as well as on-task behaviors (Ruhland and Lange, 2021). However, when Seiffer et al. investigated the efficacy of PA on children with ADHD, they observed that moderate-to-vigorous PA (MVPA) had only a small effect on total ADHD core symptoms (Seiffer et al., 2021). Another study also found that there were only small improvements in inhibitory control when PA was combined with cognitive training patterns (Dhir et al., 2021).

Adherence to PA is one of the lifestyle recommendations for ADHD children and adolescents. It was linked to fewer physician visits in a longitudinal study with 3,436 participants (Loewen et al., 2020). Pagani and co-authors showed in a study with 758 girls and 733 boys that participating in an extracurricular sport program between ages 6 and 10 was a significant predictor for lower ADHD symptoms at the age of 12 years in girls, but not in boys (Pagani et al., 2020). In a longitudinal study of a community sample of 232 monozygotic twin pairs, greater weekly energy expenditure in terms of activities in adolescence was significantly associated with lower levels of ADHD symptoms in early adulthood (Rommel et al., 2015). Higher levels of PA or engaging in PA were not related to conduct and behavioral problems, anxiety, and ADHD symptoms in a 3-year follow-up study of adolescents (Isaksson et al., 2020) and a prospective study (Peralta et al., 2018). Similarly, other studies did not report a significant effect of PA on ADHD symptoms or executive functions in ADHD children (Smith et al., 2019; Zang, 2019). Brandt et al. even reported that higher moderate activity at age 7 (*n* = 5,251) was positively associated with ADHD symptoms at age 14 (Brandt et al., 2021). Another study found that objectively measured PA at any intensity was associated with higher hyperactivity scores in boys and girls (Griffiths et al., 2016). Similar findings were reported in a longitudinal study using questionnaire-reported PA (Wiles et al., 2008).

Reviews and meta-analyses typically face some limitations, such as a small number of studies (Biddle et al., 2019; Schuch et al., 2019; Andermo et al., 2020; Lambez et al., 2020; Carter et al., 2021; Liang et al., 2021), differences in methodologies (Lambez et al., 2020; Vysniauske et al., 2020; Dhir et al., 2021; Seiffer et al., 2021), differences between studies in assessing PA and mental health outcomes (Biddle et al., 2019; Andermo et al., 2020; Liang et al., 2021; Ruhland and Lange, 2021; Welsch et al., 2021), studies with low or moderate quality and high risk of bias (Rodriguez-Ayllon et al., 2019; Vysniauske et al., 2020; Carter et al., 2021; Dhir et al., 2021; Seiffer et al., 2021; Welsch et al., 2021), and non-representative samples (Biddle et al., 2019; Rodriguez-Ayllon et al., 2019; Vysniauske et al., 2020; Bourke et al., 2021; Liang et al., 2021; Welsch et al., 2021). Individual studies have small and non-representative samples (Ahn et al., 2018; Bowe et al., 2021; Mercurio et al., 2021), low quality, no long-term follow-ups, and inadequate consideration of real-world implementation factors for intervention studies (Parker et al., 2019). In many longitudinal studies, there is a lack of repeated measurements of either PA or mental health outcomes (Ahn et al., 2018; Peralta et al., 2018; Bell et al., 2019; Doré et al., 2019, 2020; Kleppang et al., 2019; Hamer et al., 2020; Pagani et al., 2020; van Woudenberg et al., 2020; Bowe et al., 2021; Wu et al., 2021). Also, a focus on specific mental health outcomes (Rodriguez-Ayllon et al., 2019) and less frequent use of multidimensional measurements (Bell et al., 2019) are further limitations that should be mentioned.

Due to the heterogeneity of findings and the limitations outlined above, it has not been possible to reach a firm conclusion so far. Hence, there is still a need for methodologically sound, large-scale, representative, longitudinal studies (Kleppang et al., 2019; Bowe et al., 2021; Neville et al., 2021; Wu et al., 2021). The study presented in this article tries to overcome most of the aforementioned limitations. It has the following advantages: a large sample of children and adolescents (including boys and girls), and a measurement of PA and mental health problems at three time points over 10 years using a psychometrically valid multidimensional inventory (SDQ) for evaluating general mental health problems as well as ADHD symptoms. The results will provide further empirical evidence on the temporal relationships between PA and mental health problems. They will thus highlight possible causal pathways and will give directions for practical applications. Based on the findings, which indicated a positive effect of PA on mental health problems for children, we expected to find an effect of early PA on later mental health problems in general mental health problems and, in particular, symptoms of ADHD.

## Materials and methods

### Study design

This study is based on data from the German Health Interview and Examination Survey for Children and Adolescents (KiGGS), which was conducted by the Robert Koch Institute. The purpose and design of the KiGGS study have been described in detail in other reports (Kamtsiuris et al., 2007; Kurth et al., 2008; Hölling et al., 2012). The study complied with the EU General Data Protection Regulation (GDPR) and the Federal Data Protection Act (BDSG). Ethics for the KiGGS Baseline (No. 101/2000) and Wave 1 (No. EA2/058/09) have been approved by the Charité Universitätsmedizin Berlin's ethics committee. KiGGS Wave 2 (No. 2275-2014) was evaluated and approved by Hannover Medical School's ethics committee. Informed consent was obtained from all individual participants and/or their parents.

**Figure 1:**
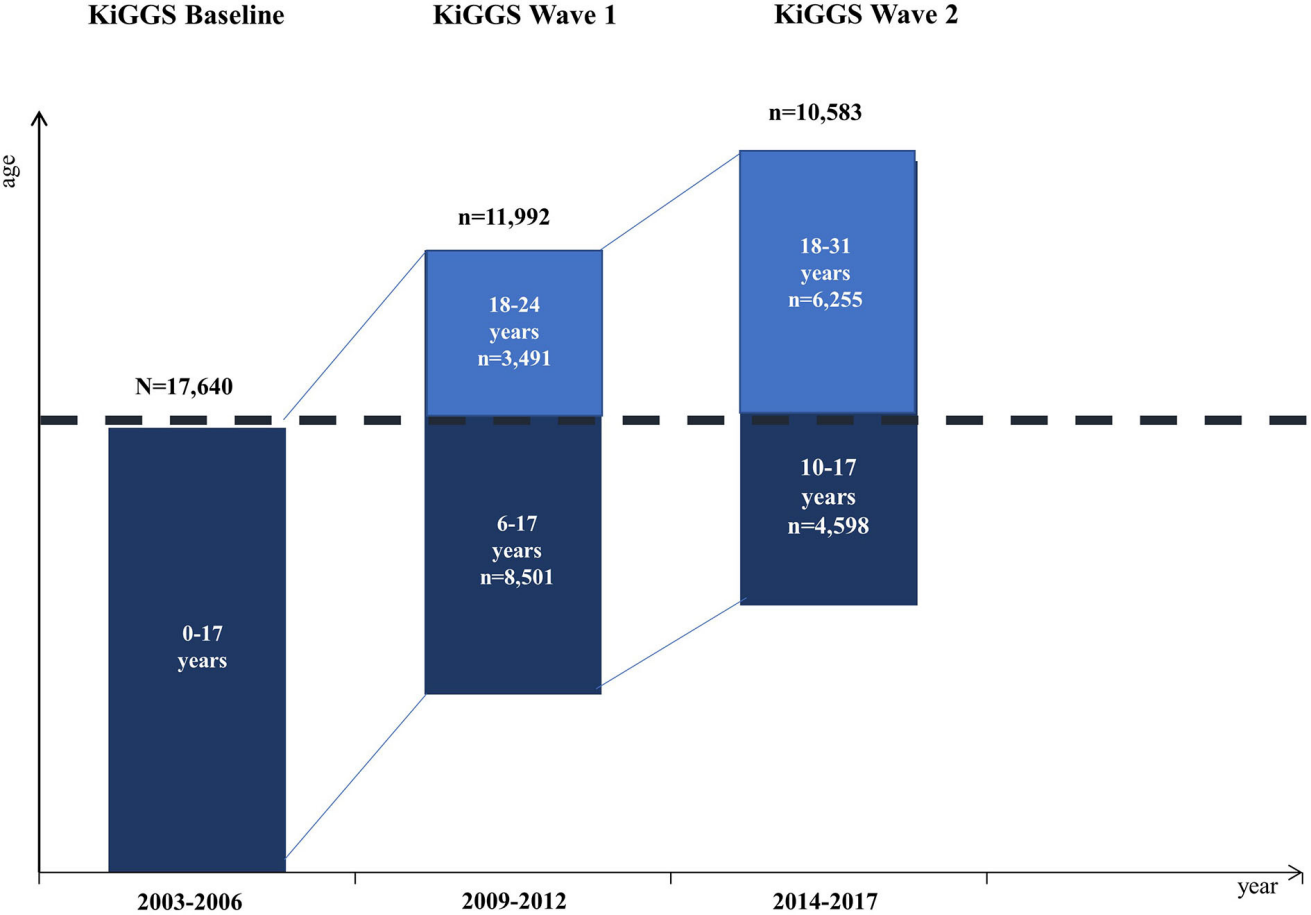
Longitudinal sample sizes of the KiGGS study. Dark blue bars: Longitudinal data from study participants not older than 17 years (the data used for analyses in this study). Light blue bars: Longitudinal data at Wave 1 and Wave 2 older than 17 years.

Data were collected in three waves, covering the intervals from 2003 to 2006 (Baseline), 2009 to 2012 (Wave 1), and 2014 to 2017 (Wave 2). At Baseline, 17,641 children and adolescents (8,656 girls and 8,985 boys) aged 0 to 17 years participated along with their parents. Participants were randomly chosen through a stratified sampling method at 167 study sites scattered throughout Germany. The response rate was 66 and 67% for boys and girls, respectively. The first follow-up survey (Wave 1) was conducted after a gap of about 6 years in the form of a telephone questionnaire survey. For this wave, 11,992 (68%) of the Baseline participants were re-invited and participated in the study (Mauz et al., 2019). In Wave 2, 15,023 children and adolescents aged between 0 and 17 years took part in a cross-sectional and longitudinal sample. In the longitudinal sample of Wave 2, there were 10,853 girls and boys (with a response rate of 61.5%) (Kurth, 2018). Figure 1 (Hoffmann et al., 2018) presents the longitudinal sample sizes of the KiGGS study.

### Assessment of physical activity

At Baseline, PA was a composite variable derived from several questions about the frequency of participation in different sports and other physical activities by children and adolescents. Data for PA were taken from parents of 0- to 10-year-old children and teenagers (11–17 years old). Three ordered categories of PA were computed from the responses, namely 1 = low, 2 = medium, and 3 = high. The WHO's recommendations (World Health Organization, 2010) for PA were taken into account for the assessments in Wave 1 and Wave 2. Parents and teenagers were asked: “How many days is your child (are you) at least 60 min physically active during a normal week?”. Responders could choose between 0 = never and 7 = all weekdays. For a better comparison between time points, three categories of PA were also defined for the responses at Waves 1 and 2 also, namely: low = never to 2 days per week, medium = 3–5 days per week, and high = 6–7 days per week. Hence, for all three time points, PA was an ordered categorical variable with three levels (low, medium, and high).

According to the study protocol, PA represents a mixed report variable, because it was assessed by parents (for 0- to 10-year-old children) and teenagers (for 11- to 17-year-old adolescents). This decision was made because adolescents are more reliable in providing behavioral information about themselves than younger children due to their matured ability to recall memories and self-perception. Moreover, researchers found a low agreement between parents' reports and adolescents' reports of PA (Sithole and Veugelers, 2008; Reichert et al., 2010; Koning et al., 2018), and agreements were usually related to organized PA (Poulain et al., 2020). Hence, the use of mixed reports of PA in this study seems to be justified.

### Mental health problems and ADHD symptoms

The KiGGS study assessed mental health by the Strength and Difficulties Questionnaire through parent- and self-report (Goodman, 1997). The SDQ is comprised of 25 items which present statements to be rated on a scale from 0 = not true to 2 = certainly true. The SDQ has five sub-scales: emotional problems, conduct problems, hyperactivity/inattention, peer problems, and prosocial behavior. The scores within these sub-scales range from 0 to 10. The total difficulties score is calculated by adding the scores of the sub-scales except for the prosocial behavior scale. A higher score indicates a higher grade of difficulties. For this study, the total difficulties score and the hyperactivity/inattention score (SDQ-H/I) taken from the parent report (for children between 3 and 17 years old) were used as an indicator for general mental health problems and symptoms of ADHD, respectively. Psychometric properties of the SDQ were investigated in normal (Klasen et al., 2000) and clinical (Becker et al., 2004) samples of German children and adolescents. The outcome scores are reliable and significantly correlated with the Child Behavior Checklist (CBCL). The questionnaire was shown to be a valid instrument to identify psychiatric patients and other disorder categories (Klasen et al., 2000; Becker et al., 2004). In this study, Cronbach's alphas for the total score of SDQ and the SDQ-H/I sub-scale were 0.80 and 0.76, respectively.

### Covariates

Parental socioeconomic status (SES) (Lampert et al., 2014) and the participant's sex, age, and body-mass index (BMI) were used as covariates in the analyses. These were chosen based on preceding studies, which indicated that they were the most important covariates (Rommel et al., 2015; Hinkley et al., 2017; Ahn et al., 2018; Bell et al., 2019; Doré et al., 2019, 2020; Kleppang et al., 2019; Schuch et al., 2019; Zhang et al., 2020; Bowe et al., 2021; Brandt et al., 2021).

### Statistical analyses

For longitudinal analyses of the bi-directional relations between PA and either mental health problems or ADHD symptoms, autoregressive cross-lagged panel models were used. All statistical analyses were conducted in R using the lavaan package (R Core Team, 2020). The pairwise deletion method was used to handle missing values. This method of handling missing data appears to be the best possible strategy, when a categorical variable (PA) is included in the analyses^1^.

**Figure 2:**
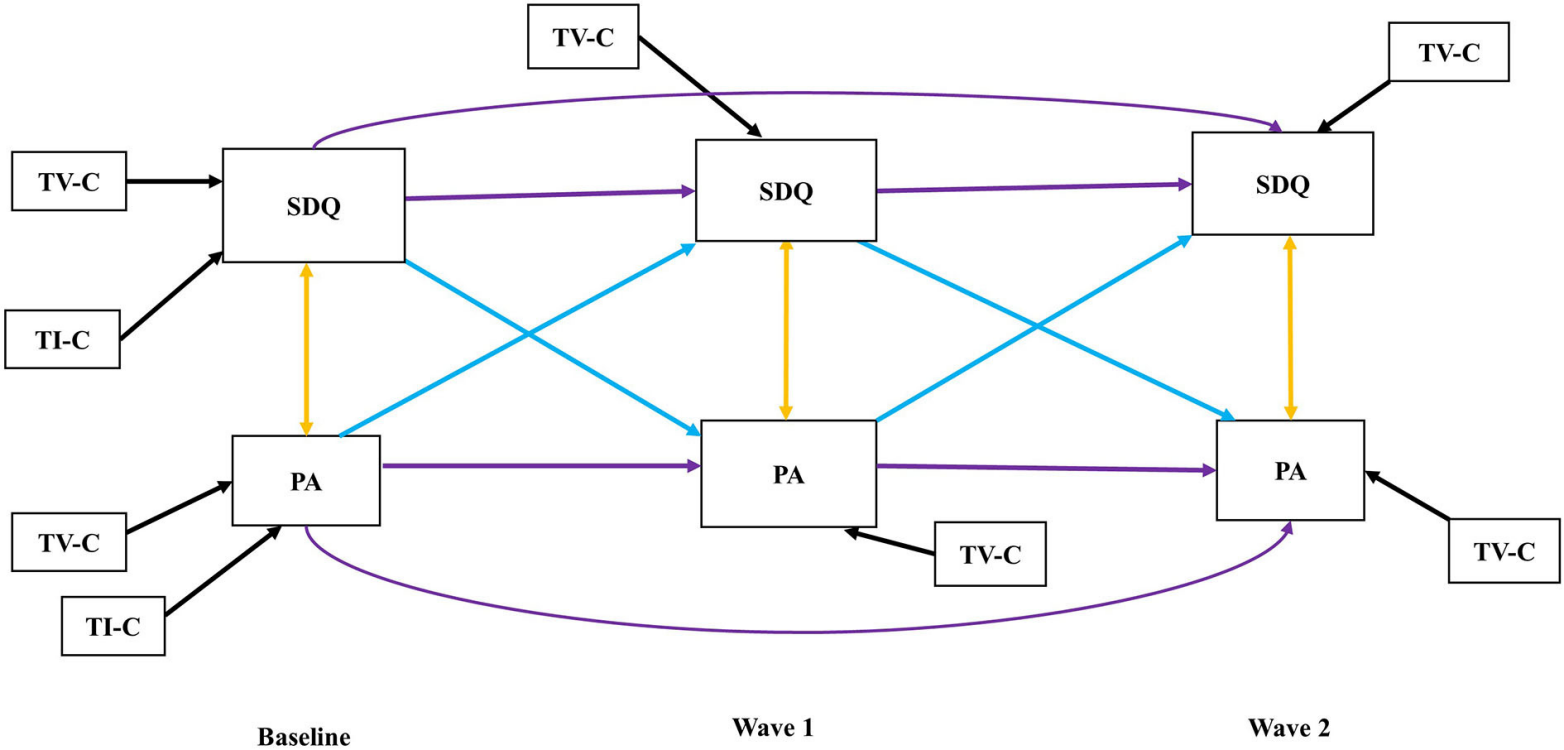
Autoregressive cross-lagged model. TV-C: Time-variant covariate, TI-C: Time-invariant covariate, Purple arrows: Auto-regressive paths, Blue arrows: Cross-lagged paths, Yellow arrows: Cross-sectional paths, Black arrows: Covariate paths, SDQ: Indicating SDQ-Total for the first model as well as SDQ-H/I for the second model at Baseline, Wave 1, and Wave 2, respectively.

Cross-lagged path models allow for the estimation of cross-lagged effects, controlling for correlations within time points, and controlling for the stability of variables over time by considering autoregressive effects. Moreover, these models can provide preliminary evidence for causality (Kearney, 2017). The cross-lagged panel model in this study includes two constructs measured at three time points (Baseline, Wave 1, and Wave 2) (Figure 2). To investigate the longitudinal reciprocal relationship between PA and general mental health using the total SDQ score and hyperactivity/inattention symptoms (SDQ-H/I), we evaluated several nested models by adding paths and controlling for parental socioeconomic status as a time-invariant and participant's body-mass index (BMI) as a time-variant covariate. Sex was used as a grouping variable. The non-significant paths were not deleted in the final model. The estimation method of the model parameters was diagonally weighted least square (DWLS). Nested models were compared using likelihood ratio tests and fit indices (CFI, TLI, and RMSEA). The robust versions of these indices were used. Because of the findings presented above, which predict the presence of cross-lagged paths and an influence of the covariates, we decided to use the full model to test the hypothesis, if the full model had an acceptable fit to the data. For testing, the statistical significance level was set to 0.05, which was adjusted to 0.025, because the coefficient for the relation between PA and SDQ, as well as PA and SDQ-H/I was tested twice (Baseline to Wave 1 and Wave 1 to Wave 2).

## Results

### Sample

The total sample at Baseline was 17,640 children and adolescents (8,654 girls and 8,986 boys) aged 0 −17 years. Due to the method used for handling missing data, the analysis was run with 17,638 participants (8,985 boys and 8,653 girls). We used data for children and adolescents up to 17 years old (see dark blue bars in Figure 1), because parent-reported SDQ data were only available up to this age. The exact numbers and descriptive characteristics of the longitudinal sample at Baseline, Wave 1, and Wave 2 are shown in Table 1. Note that the number of participants declines across the three waves due to participants dropping out. The correlation matrix for the variables at the three time points can be found in Supplementary Table 1 in Appendix A.

**Table 1:** Descriptive characteristics of the study population at Baseline, Wave 1, and Wave 2.

		***N***	**Mean**	**SD**	**Min**	**Max**
**Baseline**	
Sex	Boys	8,986 (50.9%)			1	2
	Girls	8,654 (49.1%)				
	Total	17,640				
Age (years)		17,640	8.51	5.07	0	17
SDQ-emotion		14,497	1.78	1.80	0	10
SDQ-behavioral problems		14,502	1.93	1.53	0	9
SDQ-hyperactivity inattention		14,498	3.14	2.27	0	10
SDQ-peer problems		14,496	1.44	1.61	0	8
SDQ-prosocial behavior		14,529	7.80	1.72	1	10
SDQ-Total		14,477	8.29	5.15	0	32
Physical activity	Low	6,005 (36.1%)			1	3
	Medium	5,422 (32.6%)				
	High	5,221 (29.6%)				
	Total	16,648				
Socioeconomic status		17,306	11.65	3.84	3	21
Body-mass index (kg/m^2^)		17,493	18.23	3.64	10.84	33.08
**Wave 1**	
Age (years)		8,501	11.73	3.385	6	17
SDQ-emotion		8,441	1.95	1.84	0	9
SDQ-behavioral problems		8,442	1.94	1.52	0	9
SDQ-hyperactivity inattention		8,440	2.94	2.18	0	10
SDQ-peer problems		8,439	1.29	1.49	0	8
SDQ-prosocial behavior		8,440	8.35	1.55	0	10
SDQ-Total		8,436	8.12	4.96	0	31
Physical activity	Low	1,694 (20.8%)			1	3
	Medium	4,105 (50.4%)				
	High	2,351 (28.8%)				
	Total	8,150				
Body-mass index (kg/m^2^)		7,234	18.49	3.66	10.27	34.89
**Wave 2**	
Age (years)		4,598	14	1.99	10	17
SDQ-emotion		4,508	1.70	1.85	0	10
SDQ-behavioral problems		4,512	1.54	1.43	0	8
SDQ-hyperactivity inattention		4,511	2.57	2.06	0	10
SDQ-peer problems		4,507	1.32	1.60	0	8
SDQ-prosocial behavior		4,508	8.01	1.73	1	10
SDQ-Total		4,502	7.13	4.90	0	28
Physical activity	Low	1,214 (27.8%)			1	3
	Medium	2,173 (49.8%)				
	High	980 (22.4%)				
	Total	4,367				
Body-mass index (kg/m^2^)		3,592	20.84	4.006	12.06	40.025

### Longitudinal autoregressive cross-lagged model

#### Cross-lagged models for the relationship of PA and SDQ-Total

Fit indices of the three nested models can be found in Table 2. The model fit indices of Model 3 were good (CFI = 0.95, TLI = 0.90, RSMEA = 0.031). The fit indices of Model 2, which excludes the covariates, were almost perfect (CFI > 0.99, TLI = 0.99, RSMEA = 0.008). Nevertheless, we continued with Model 3, which includes all cross-lagged paths and the covariates, because of our hypothesis and previous research, which found the covariates to be relevant. In Table 3, the results for the statistically significant path coefficients of Model 3 including autoregressive paths, adjacent cross-lagged paths, and covariates are shown.

**Table 2:** Models of the reciprocal relations between PA and SDQ-Total.

**Model**		χ^**2**^		***df***	***p***	**CFI**	**TLI**	**RMSEA [90% CI]**
		**Boys**	**Girls**					
Model 1	Autoregressive model with adjacent and distant paths	29.052	23.041	12	0.001	0.992	0.98	0.020 [0.014–0.025]
Model 2	Adding adjacent cross-lagged paths	2.862	3.169	4	0.334	1	0.99	0.008 [0.00–0.019]
Model 3	Adding covariates paths	190.306	154.802	36	0.000	0.95	0.90	0.031 [0.028–0.034]

**Table 3:** Significant paths coefficients of Model 3 between PA and SDQ-Total.

**Group**	**Boys**	**Girls**
**Paths from…to…**.	**Beta (Std.Err)/P value**	**Beta (Std.Err)/P value**
Autoregressive paths		
SDQ0 to SDQ2	0.225 (0.027)^**^	0.165 (0.025)^**^
SDQ0 to SDQ1	0.548 (0.013)^**^	0.536 (0.013)^**^
SDQ1 to SDQ2	0.424 (0.021)^**^	0.478 (0.20)^**^
PA0 to PA1	0.049 (0.021)^+^	−0.004 (0.020)
PA1 to PA2	0.254 (0.026)^**^	0.161 (0.029)^**^
Cross-lagged paths		
PA0 to SDQ1	−0.329 (0.091)^**^	−0.233 (0.085)^*^
SDQ0 to PA1	−0.012 (0.004)^*^	−0.006 (0.004)
SDQ1 to PA2	−0.009 (0.005)	−0.011 (0.005)
Cross-sectional paths		
SDQ0 to PA0	−0.549 (0.070)^**^	−0.295 (0.063)^**^
SDQ1 to PA1	−0.164 (0.077) ^+^	0.060 (0.072)
SDQ2 to PA2	−0.276 (0.103)^*^	−0.120 (0.092)

PA0 and SDQ0 = measured at Baseline, PA1 and SDQ1 = measured at Wave 1, and PA2 and SDQ2 = measured at Wave 2.

** p < 0.001,

* p < 0.01,

+ p < 0.05.

Among boys, the autoregressive paths between adjacent and distant time points were significant for SDQ and for adjacent paths also for PA. The stability of mental health problems decreased for the autoregressive paths over time (beta_Baseline−Wave1_ = 0.548 to beta_Wave1−Wave2_ = 0.424 for SDQ), whereas the stability of PA increased for the autoregressive paths over time (beta_Baseline−Wave1_ = 0.049 to beta_Wave1−Wave2_ = 0.254) (Table 3). The cross-sectional correlations between PA and SDQ were negative and significant (*r*s ranged from −0.549 to −0.276).

Among girls, the autoregressive paths between adjacent and distant time points were significant for SDQ and PA (only one adjacent path). The stability of mental health problems decreased slightly for the adjacent autoregressive paths over time (beta_Baseline−Wave1_ = 0.536 to beta_Wave1−Wave2_ = 0.478) for SDQ, whereas the stability of PA increased for the autoregressive paths over time (beta_Baseline−Wave1_ = −0.004 to beta_Wave1−Wave2_ = 0.161) (Table 3). The cross-sectional correlation between PA and SDQ was only significant at Baseline (*r*s = −0.295).

The cross-lagged path from PA at Baseline to SDQ at Wave 1 was significant and negative for boys and girls (beta = −0.329 and beta = −0.233). Thus, higher PA at Baseline was associated with lower mental health problems score at Wave 1. Moreover, reciprocally, there was a negative significant association between SDQ at Baseline with PA at Wave 1 (beta = −0.012, *p* < 0.01) among boys. Considering the adjusted significance level of 0.025, the association between SDQ at Wave 1 and PA at Wave 2 (beta = −0.011, *P*-value = 0.043) was not significant among girls.

Based on Model 3, estimated means of the total score of the SDQ and their 90% confidence intervals were computed. These are shown in Table 4. These means illustrate the positive effect of PA on both boys and girls.

**Table 4:** Estimated means of SDQ-Total at Wave 1 in different levels of PA at baseline.

	**Boys**	**Girls**
	**SDQ-Total mean (SE) [90% CI]**	
Physical activity		
Low	8.79 (0.17) [8.44–9.14]	7.78 (0.16) [7.47–8.10]
Medium	8.49 (0.13) [8.22–8.76]	7.51 (0.12) [7.26–7.75]
High	8.24 (0.12) [7.99–8.49]	7.39 (0.13) [7.12–7.66]

#### Cross-lagged models for the relationship of PA and SDQ-H/I

Table 5 presents fit indices for the three nested models calculated to investigate the longitudinal relationship between PA and ADHD symptoms, as determined by the SDQ-H/I scores. Model 3 included all potential paths, and its model fit indices were considered to be good. The results of this model including autoregressive paths, adjacent cross-lagged paths, and covariates are reported in Table 6.

**Table 5:** Models of the reciprocal relations between PA and SDQ-H/I.

**Model**		χ^**2**^		***df***	***p***	**CFI**	**TLI**	**RMSEA [90% CI]**
		**Boy**	**Girl**					
Model 1	Autoregressive model with adjacent and distant paths	27.539	24.474	12	0.000	0.989	0.973	0.020 [0.014–0.025]
Model 2	Adding adjacent cross-lagged paths	4.451	3.043	4	0.22	0.999	0.993	0.010 [0.000–0.021]
Model 3	Adding covariates paths	208.849	192.844	36	0.000	0.933	Robust: 0.855,	0.034 [0.031–0.037]
							Standard: 0.916	

**Table 6:** Significant paths coefficients of Model 3 between PA and SDQ-H/I.

**Group**	**Boys**	**Girls**
**Paths from…to…**.	**Beta (Std.Err)/P value**	**Beta (Std.Err)/P value**
Autoregressive paths		
SDQ-H/I 0 to SDQ-H/I 2	0.119 (0.026)^**^	0.099 (0.027)^**^
SDQ-H/I 0 to SDQ-H/I 1	0.521 (0.014)^**^	0.519 (0.014)^**^
SDQ-H/I 1 to SDQ-H/I 2	0.495 (0.022)^**^	0.457 (0.021)^**^
PA1 to PA2	0.259 (0.026)^**^	0.165 (0.029)^**^
PA0 to PA1	0.056 (0.021)^*^	0.00 (0.20)
Cross-lagged paths		
PA0 to SDQ-H/I 1	−0.188 (0.04)^**^	−0.152 (0.037)^**^
PA 1 to SDQ-H/I 2	0.103 (0.051)	0.017 (0.046)
SDQ-H/I 1 to PA2	−0.009 (0.012)	−0.029 (0.012) ^+^
Cross-sectional paths		
SDQ-H/I 0 to PA0	−0.067 (0.031)^+^	0.023 (0.028)
SDQ-H/I 1 to PA1	0.025 (0.036)	0.109 (0.031)^**^
SDQ-H/I 2 to PA2	0.019 (0.043)	0.095 (0.038)^+^

PA0 and SDQ-H/I 0 = measured at Baseline, PA1 and SDQ-H/I 1 = measured at Wave 1, and PA2 and SDQ-H/I 2 = measured at Wave 2.

** p < 0.001,

* p < 0.01,

+ p < 0.05.

For boys, the autoregressive paths between adjacent and distant time points were significant for hyperactivity/inattention and PA (only in adjacent paths). The stability of SDQ-H/I decreased slightly for the autoregressive paths over time (beta_Baseline−Wave1_ = 0.521 to beta_Wave1−Wave2_ = 0.495) for SDQ-H/I, whereas the stability of PA increased for the autoregressive paths over time (beta_Baseline−Wave1_ = 0.056 to beta_Wave1−Wave2_ = 0.259) (Table 6). The cross-sectional correlation between PA and SDQ-H/I was only significant at Baseline (*r*s = −0.067).

For girls, the autoregressive paths between adjacent and distant time points were significant for SDQ-H/I and PA (only in one adjacent path). The stability of SDQ-H/I symptoms decreased for the autoregressive paths over time (beta_Baseline−Wave1_ = 0.519 to beta_Wave1−Wave2_ = 0.457), whereas the stability of PA increased for the autoregressive paths over time (beta_Baseline−Wave1_ < 0.001 to beta_Wave1−Wave2_ = 0.165) (Table 6). For girls, there was a small positive cross-sectional correlation between PA and SDQ-H/I symptoms at Wave 1 and Wave 2 (*r*s = 0.109, *p* < 0.01 and *r*s = 0.095, *p* < 0.05, respectively).

The cross-lagged path from PA at Baseline to SDQ-H/I at Wave 1 was significant and negative for boys and girls alike (beta = −0.188 and beta = −0.152). Thus, a higher PA at Baseline was associated with lower symptoms of hyperactivity/inattention at Wave 1. Interestingly, the estimate of the cross-lagged path from PA at Wave 1 to SDQ-H/I at Wave 2 was positive among boys and girls (beta = 0.103, *P*-value = 0.043, beta = 0.017, *P*-value = 0.707). There was a negative significant association between SDQ-H/I at Wave 1 with PA at Wave 2, but only among girls (beta = −0.029, *p* < 0.05). The estimated means of SDQ-H/I are reported for the three categories of PA for cross-lagged path from PA at Baseline and Wave 1 to SDQ-H/I at Wave 1 and Wave 2.

Table 7 shows the means and the 95%-confidence intervals predicted from Model 3. For boys, the estimated means of SDQ-H/I at Wave 1 were higher for a low level of PA. For girls, the estimated mean is lower for a high level of PA. At Wave 2, the relation was reversed for boys. The estimated mean SDQ-H/I was lower for low PA. The sample size used was based on the estimated model (Table 7).

**Table 7:** Estimated means of SDQ-H/I for different levels of PA.

**Means of SDQ-H/I**			**Means of SDQ-H/**
**at Wave 1 for**			**I at Wave 2 for**
**the three levels of**			**the three levels**
**PA at Baseline**			**of PA at Wave 1**
	**Boys**	**Girls**	**Boys**
	**SDQ-H/I mean (SE)**		**SDQ-H/I mean (SE)**
	**[95% CI]**		**[95% CI]**
Physical activity	
Low	3.33 (0.07)	2.51 (0.06)	2.34 (0.14)
	[3.18–3.49]	[2.38–2.64]	[2.06–2.63]
Medium	3.20 (0.06)	2.48 (0.05)	2.69 (0.08)
	[3.08–3.32]	[2.38–2.58]	[2.52–2.85]
High	3.21 (0.05)	2.37 (0.05)	2.88 (0.101)
	[3.10–3.32]	[2.26–2.49]	[2.68–3.08]

## Discussion

Overcoming several limitations of earlier studies, this study is based on a large, longitudinal panel study of children and adolescents enrolled in the German KiGGS study (Kamtsiuris et al., 2007). Data from this study allowed us to examine the reciprocal relationship between PA and general mental health problems, as well as ADHD symptoms. An autoregressive cross-lagged panel model was applied for the three time points investigated: Baseline, Wave 1, and Wave 2. After a control for autoregressive paths, previous mental health problems, and time-variant and time-invariant covariates, PA showed a significant negative relationship with subsequent general mental health problems and ADHD symptoms. Evidence was found for PA at Baseline as a predictor of general mental health problems, as well as ADHD symptoms at Wave 1, that is after about 6 years.

### Longitudinal relationship between PA and general developmental psychopathology

In line with other studies (Doré et al., 2019; Loewen et al., 2019; Gómez-Baya et al., 2020; Wu et al., 2021), the findings indicated that higher levels of PA in childhood can predict mental health problems 6 years later in both boys and girls. This confirms a general tendency found in several studies with prepubertal children (Hamer et al., 2020). However, for the cross-lagged association from Wave 1 to Wave 2 (i.e., during adolescence), there was no significant path from PA to mental health problems neither for boys nor girls. Only the latter finding is inconsistent with studies that investigated longitudinal relationships between PA and later mental health problems among adolescents (Bell et al., 2019; Doré et al., 2020; Gómez-Baya et al., 2020; Isaksson et al., 2020; Kandola et al., 2020; Bowe et al., 2021). All these studies found that inactivity or low activity was associated with increased emotional difficulties, depressive symptoms (Bell et al., 2019; Gómez-Baya et al., 2020; Kandola et al., 2020; Bowe et al., 2021), and a negative outcome for autonomy, competence, and relatedness perceptions (Doré et al., 2020). The effect of early PA on mental health in the teenage years may be moderated by psychosomatic and psychosocial puberty issues, personality identification, peer problems, BMI development, and a change in sedentary behavior. The samples used in different studies may have deviated from each other with respect to these factors. It is a challenge to control all these factors.

There were significant effects from total SDQ scores at Baseline to PA at Wave 1 among boys. In line with these results, a study found that mental health problems in childhood may be a risk factor for lower preadolescent activity among boys (Nigg et al., 2021). The path coefficients, however, were clearly smaller than the path coefficients from PA to SDQ-Total, which indicates that PA is more predictive of general mental health problems than inversely general mental health problems for PA.

Significant high stability in autoregressive paths was found for both SDQ-Total and PA from Baseline to Wave 2. Adjacent and distant paths were significant for SDQ-Total. This may indicate that individual differences in mental health problems increase over a 10-year period from preschool to adolescence for both sexes reflecting developmental changes from childhood to adolescence. In contrast, only adjacent paths were significant for PA from Baseline to Wave 1 among boys and Wave 1 to Wave 2 in boys and girls. This might be due to different measurements of PA at Baseline and the two subsequent waves (see Limitations).

In summary, the findings provide new evidence for a positive relationship between early PA and later mental health, which may indicate a positive causal relationship between them. PA may be a viable predictor and a preventive factor of mental health problems in children and adolescents.

### Longitudinal relationship between PA and ADHD symptoms

In this study, we observed a temporal and reciprocal relationship between PA and ADHD symptoms. Higher levels of PA in childhood were associated with lower ADHD symptoms 6 years later for boys and girls. This finding is consistent with previous studies which found positive effects of PA on the reduction of ADHD symptoms (Rommel et al., 2015; Loewen et al., 2020; Pagani et al., 2020; Liang et al., 2021) and cognitive performance related to ADHD (Biddle et al., 2019; Lambez et al., 2020; Ruhland and Lange, 2021) among children and adolescents. In this study, the cross-lagged relationship between PA and ADHD symptoms was positive from Wave 1 to Wave 2 among boys and girls. It implies that a higher level of PA was associated with higher levels of ADHD symptoms. Although the effect size was small, other studies have reported similar results (Ganjeh et al., 2021). Three other longitudinal studies found that a 15–30 min increase in PA was associated with higher SDQ-H/I scores (Ahn et al., 2018) and that more PA predicted more ADHD symptoms (Wiles et al., 2008; Brandt et al., 2021). Differentiating between the frequency, duration, and intensity of PA, as well as adding other covariates to the analysis may explain the different findings (Wiles et al., 2008; Brandt et al., 2021; Ganjeh et al., 2021).

Notably, significant cross-lagged effects from ADHD symptoms to PA were found only among girls from Wave 1 to Wave 2. This means that girls with higher levels of ADHD symptoms had lower levels of PA about 4 years later. However, there was no reciprocal path, from PA to ADHD symptoms for girls for these waves. This finding may suggest that PA should be encouraged for teenage girls with severe ADHD to support their mental health. However, more research is required.

Concerning stability, adjacent and distant autoregressive paths were significant and decreased ADHD symptoms. This means that symptoms of this disorder can change during developmental periods, as is known from hyperactivity (Banaschewski et al., 2017). The autoregressive paths of PA were significant only for adjacent paths. The increasing path strengths of PA in the model for ADHD imply that PA has changed little over the 10-year period, specifically among boys. This might be a result of different measurements of PA at Baseline and at the two following waves (see Limitations). However, it seems that other models, for example, growth curve models, may more clearly explain the trajectories of changes over time than does our model. Hence, it is suggested that further studies should investigate trajectories of PA and mental health problems.

## Strengths and limitations

The longitudinal approach with a large sample size of children and adolescents is a strength of this study because it can help to gain information about the causal relationship between PA and general mental health problems, as well as ADHD symptoms. Using SDQ as a valid multidimensional inventory to evaluate not only the general mental health problems but also ADHD symptoms is another strength of this study. Our sample covers a broad age range from preschool children to adolescents. Furthermore, evaluating PA, general mental health problems, and ADHD symptoms at three time points allows controlling for previous values of these variables in the longitudinal models, thus creating stronger evidence.

Despite the strengths of this study, which overcome some of the shortcomings of previous works, the study did have some limitations. At Wave 1 and Wave 2, we used one core-question to evaluate PA according to the WHO recommendations. Therefore, we had no insight into the physical activities performed (e.g., the type of activity or its intensity). In consequence, we suggest that future studies may use questionnaires and/or objective methods to obtain more detailed information about the kind of PA. Furthermore, PA at Baseline was a composite variable derived from several other more specific variables and was therefore not identical to Wave 1 and Wave 2. This may have contributed to the rather low stability of PA between Baseline and Wave 1 and the substantially higher stability between Wave 1 and 2. Using a mixed report variable of PA, namely parental reports for children up to 11 years of age and self-reports for older children, is another limitation. This decision was justified by findings of low agreement between parent report and self-report of PA for adolescents (Sithole and Veugelers, 2008; Reichert et al., 2010; Koning et al., 2018). There is, however, no direct evidence that a mixed report increases the validity of the measurement of PA. Another limitation was the exclusive use of parent-reported data on mental health problems, although this was methodologically justified for developmental reasons. Finally, our results are not fully representative of the German population because we did not use the appropriate weighting of factors in the analyses.

## Conclusion

The findings of this study provide reliable evidence for the hypothesis that PA has positive effects on later mental health problems, including ADHD symptoms in children but not in adolescents. A higher level of PA at preschool age may lead to lower SDQ scores and ADHD symptoms 6 years later. The effects were stronger for boys than for girls. In conclusion, the findings suggest that weekly medium-to-high PA may be helpful to prevent mental health problems (including ADHD symptoms) in children.

## Data availability statement

The raw data supporting the results of this article will be made available by the authors, without undue reservation.

## Ethics statement

The studies involving human participants were reviewed by the responsible Ethics Committees. The approval of Robert Koch Institute studies' data protection was received and the compliance of all ethics with the data protection provisions set out in the EU General Data Protection Regulation (GDPR) and the Federal Data Protection Act (BDSG) was confirmed. KiGGS Baseline (No. 101/2000) and Wave 1 (No. EA2/058/09) ethics have been assessed by Charité-Universitätsmedizin Berlin's Ethics Committee. KiGGS Wave 2 (No. 2275-2014) was also evaluated and approved by Hannover Medical School's Ethics Committee. Informed consent was obtained from all individual participants and/or their parents included in the study. Written informed consent to participate in this study was provided by the participants' legal guardian/next of kin.

## Author contributions

PG performed the study conception, formal analysis, and the writing of the original draft. PG and YH contributed to the methodology of the study. PG and AR contributed to interpreting the results. Writing—review and editing were conducted by PG, AR, YH, TM, NS, UR-S, RK, and AB. All authors contributed to the article and approved the submitted version.

## Conflict of interest

The authors declare that the research was conducted in the absence of any commercial or financial relationships that could be construed as a potential conflict of interest.

## Publisher's note

All claims expressed in this article are solely those of the authors and do not necessarily represent those of their affiliated organizations, or those of the publisher, the editors and the reviewers. Any product that may be evaluated in this article, or claim that may be made by its manufacturer, is not guaranteed or endorsed by the publisher.

## Footnotes

1https://rdrr.io/cran/lavaan/man/lavOptions.html

## References

[B1] Ahn J. V. Sera F. Cummins S. Flouri E. (2018). Associations between objectively measured physical activity and later mental health outcomes in children: findings from the UK Millennium Cohort Study. J. Epidemiol. Community Health 72, 94–100. 10.1136/jech-2017-209455 29183954

[B2] American Psychiatric Association (2016). Diagnostic and Statistical Manual of Mental Disorders, 5th Edn. Arlington, VA: American Psychiatric Association.

[B3] Andermo S. Hallgren M. Nguyen T. T. D. Jonsson S. Petersen S. Friberg M.. (2020). School-related physical activity interventions and mental health among children: a systematic review and meta-analysis. Sports Med. Open 6, 25. 10.1186/s40798-020-00254-x 32548792 PMC7297899

[B4] Banaschewski T. Becker K. Döpfner M. Holtmann M. Rösler M. Romanos M. (2017). Attention-deficit/hyperactivity disorder. Dtsch. Ärztebl. Int. 114, 149–159. 10.3238/arztebl.2017.0149 28351467 PMC5378980

[B5] Becker A. Woerner W. Hasselhorn M. Banaschewski T. Rothenberger A. (2004). Validation of the parent and teacher SDQ in a clinical sample. Eur. Child. Adolesc. Psychiatry 13(Suppl. 2), II11–II16. 10.1007/s00787-004-2003-5 15243781

[B6] Bélair M. A. Kohen D. E. Kingsbury M. Colman I. (2018). Relationship between leisure time physical activity, sedentary behavior and symptoms of depression and anxiety: evidence from a population-based sample of Canadian adolescents. BMJ Open 8, e021119. 10.1136/bmjopen-2017-021119 30337306 PMC6196847

[B7] Bell S. L. Audrey S. Gunnell D. Cooper A. Campbell R. (2019). The relationship between physical activity, mental wellbeing and symptoms of mental health disorder in adolescents: a cohort study. Int. J. Behav. Nutr. Phys. Act. 16, 138. 10.1186/s12966-019-0901-7 31878935 PMC6933715

[B8] Biddle S. J. Ciaccioni S. Thomas G. Vergeer I. (2019). Physical activity and mental health in children and adolescents: an updated review of reviews and an analysis of causality. Psychol. Sport Exerc. 42, 146–155. 10.1016/j.psychsport.2018.08.011

[B9] Bourke M. Hilland T. A. Craike M. (2021). A systematic review of the within-person association between physical activity and affect in children's and adolescents' daily lives. Psychol. Sport Exerc. 52, 101825. 10.1016/j.psychsport.2020.101825

[B10] Bowe A. K. Healy C. Cannon M. Codd M. B. (2021). Physical activity and emotional-behavioural difficulties in young people: a longitudinal population-based cohort study. Eur. J. Public Health 31, 167–173. 10.1093/eurpub/ckaa182 33176354

[B11] Brandt V. Patalay P. Kerner Auch Koerner J. (2021). Predicting ADHD symptoms and diagnosis at age 14 from objective activity levels at age 7 in a large UK cohort. Eur. Child Adolesc. Psychiatry 30, 877–884. 10.1007/s00787-020-01566-9 32506264 PMC8140967

[B12] Brown H. E. Pearson N. Braithwaite R. E. Brown W. J. Biddle S. J. H. (2013). Physical activity interventions and depression in children and adolescents: a systematic review and meta-analysis. Sports Med. 43, 195–206. 10.1007/s40279-012-0015-8 23329611

[B13] Brylka A. Wolke D. Ludyga S. Bilgin A. Spiegler J. Trower H.. (2021). Physical activity, mental health, and well-being in very pre-term and term born adolescents: an individual participant data meta-analysis of two accelerometry studies. Int. J. Environ. Res. Public Health 18, 1735. 10.3390/ijerph18041735 33579022 PMC7916780

[B14] Carter T. Pascoe M. Bastounis A. Morres I. D. Callaghan P. Parker A. G. (2021). The effect of physical activity on anxiety in children and young people: a systematic review and meta-analysis. J. Affect. Disord. 285, 10–21. 10.1016/j.jad.2021.02.026 33618056

[B15] Cerrillo-Urbina A. J. García-Hermoso A. Sánchez-López M. Pardo-Guijarro M. J. Santos Gómez J. L. Martínez-Vizcaíno V. (2015). The effects of physical exercise in children with attention deficit hyperactivity disorder: a systematic review and meta-analysis of randomized control trials. Child Care Health Dev. 41, 779–788. 10.1111/cch.12255 25988743

[B16] Dhir S. Teo W. P. Chamberlain S. R. Tyler K. Yücel M. Segrave R. A. (2021). The effects of combined physical and cognitive training on inhibitory control: a systematic review and meta-analysis. Neurosci. Biobehav. Rev. 128, 735–748. 10.1016/j.neubiorev.2021.07.008 34256070 PMC7611490

[B17] Doering S. Lichtenstein P. Gillberg C. Middeldorp C. M. Bartels M. Kuja-Halkola R.. (2019). Anxiety at age 15 predicts psychiatric diagnoses and suicidal ideation in late adolescence and young adulthood: results from two longitudinal studies. BMC Psychiatry 19, 363. 10.1186/s12888-019-2349-3 31727035 PMC6857289

[B18] Doré I. Sabiston C. M. Sylvestre M. P. Brunet J. O'Loughlin J. Nader P. A.. (2019). Years participating in sports during childhood predicts mental health in adolescence: a 5-year longitudinal study. J. Adolesc. Health 64, 790–796. 10.1016/j.jadohealth.2018.11.024 31122508

[B19] Doré I. Sylvester B. Sabiston C. Sylvestre M. P. O'Loughlin J. Brunet J.. (2020). Mechanisms underpinning the association between physical activity and mental health in adolescence: a 6-year study. Int. J. Behav. Nutr. Phys. Act. 17, 9. 10.1186/s12966-020-0911-5 32005251 PMC6993479

[B20] Erickson K. I. Hillman C. Stillman C. M. Ballard R. M. Bloodgood B. Conroy D. E.. (2019). Physical activity, cognition, and brain outcomes: a review of the 2018 physical activity guidelines. Med. Sci. Sports Exerc. 51, 1242–1251. 10.1249/MSS.0000000000001936 31095081 PMC6527141

[B21] Erskine H. E. Moffitt T. E. Copeland W. E. Costello E. J. Ferrari A. J. Patton G.. (2015). A heavy burden on young minds: the global burden of mental and substance use disorders in children and youth. Psychol. Med. 45, 1551–1563. 10.1017/S0033291714002888 25534496 PMC5922255

[B22] Erskine H. E. Norman R. E. Ferrari A. J. Chan G. C. K. Copeland W. E. Whiteford H. A.. (2016). Long-term outcomes of attention-deficit/hyperactivity disorder and conduct disorder: a systematic review and meta-analysis. J. Am. Acad. Child Adolesc. Psychiatry 55, 841–850. 10.1016/j.jaac.2016.06.016 27663939

[B23] Ganjeh P. Meyer T. Hagmayer Y. Kuhnert R. Ravens-Sieberer U. Steinbuechel N.. (2021). Physical activity improves mental health in children and adolescents irrespective of the diagnosis of attention-deficit/hyperactivity disorder (ADHD) - a multi-wave analysis using data from the KiGGS study. Int. J. Environ. Res. Public Health 18, 2207. 10.3390/ijerph18052207 33668090 PMC7967688

[B24] Göbel K. Baumgarten F. Kuntz B. Hölling H. Schlack R. (2018). ADHD in children and adolescents in Germany. Results of the cross-sectional KiGGS Wave 2 study and trends. J. Health Monit. 3, 42–49. 10.17886/RKI-GBE-2018-085 35586800 PMC8848912

[B25] Gómez-Baya D. Calmeiro L. Gaspar T. Marques A. Loureiro N. Peralta M.. (2020). Longitudinal association between sport participation and depressive symptoms after a two-year follow-up in mid-adolescence. Int. J. Environ. Res. Public Health 17. 10.3390/ijerph17207469 33066534 PMC7602134

[B26] Goodman R. (1997). The Strengths and Difficulties Questionnaire: a research note. J. Child Psychol. Psychiatry 38, 581–586. 10.1111/j.1469-7610.1997.tb01545.x 9255702

[B27] Griffiths L. Geraci M. Cortina-Borja M. Sera F. Law C. Joshi H.. (2016). Associations between children's behavioural and emotional development and objectively measured physical activity and sedentary time: findings from the UK Millennium Cohort Study. Longitud. Life Course Stud. 7, 124–143. 10.14301/llcs.v7i2.353

[B28] Groß S. Figge C. Matthies S. Philipsen A. (2015). ADHS im Erwachsenenalter: Diagnostik und Therapie. Nervenarzt 86, 1171–1178. 10.1007/s00115-015-4328-3 26341837

[B29] Halperin J. M. Berwid O. G. O'Neill S. (2014). Healthy body, healthy mind? The effectiveness of physical activity to treat ADHD in children. Child. Adolesc. Psychiatr. Clin. N. Am. 23, 899–936. 10.1016/j.chc.2014.05.005 25220093

[B30] Hamer M. Patalay P. Bell S. Batty G. D. (2020). Change in device-measured physical activity assessed in childhood and adolescence in relation to depressive symptoms: a general population-based cohort study. J. Epidemiol. Community Health 74, 330–335. 10.1136/jech-2019-213399 31974294

[B31] Heinze K. Cumming J. Dosanjh A. Palin S. Poulton S. Bagshaw A. P.. (2021). Neurobiological evidence of longer-term physical activity interventions on mental health outcomes and cognition in young people: a systematic review of randomised controlled trials. Neurosci. Biobehav. Rev. 120, 431–441. 10.1016/j.neubiorev.2020.10.014 33172601

[B32] Hinkley T. Timperio A. Salmon J. Hesketh K. (2017). Does preschool physical activity and electronic media use predict later social and emotional skills at 6 to 8 years? A cohort study. J. Phys. Act. Health 14, 308–316. 10.1123/jpah.2015-0700 28169562

[B33] Hoffmann R. Lange M. Butschalowsky H. Houben R. Schmich P. Allen J.. (2018). Querschnitterhebung von KiGGS Welle 2 – Teilnehmendengewinnung, Response und Repräsentativität. J. Health Monit. 3, 82–96. 10.17886/RKI-GBE-2018-015

[B34] Hölling H. Schlack R. Kamtsiuris P. Butschalowsky H. Schlaud M. Kurth B. M. (2012). Die KiGGS-studie. Bundesweit repräsentative Längs- und Querschnittstudie zur Gesundheit von Kindern und Jugendlichen im Rahmen des Gesundheitsmonitorings am Robert Koch-Institut. Bundesgesundheitsblatt Gesundheitsforschung Gesundheitsschutz 55, 836–842. 10.1007/s00103-012-1486-3 22736165

[B35] Isaksson J. Selinus E. N. Åslund C. Nilsson K. W. (2020). Physical activity in early adolescence predicts depressive symptoms 3 years later: a community-based study. J. Affect. Disord. 277, 825–830. 10.1016/j.jad.2020.09.008 33065823

[B36] Kamtsiuris P. Lange M. Schaffrath Rosario A. (2007). Der Kinder- und Jugendgesundheitssurvey (KiGGS): Stichprobendesign, Response und Nonresponse-Analyse. Bundesgesundheitsblatt Gesundheitsforschung Gesundheitsschutz 50, 547–556. 10.1007/s00103-007-0215-9 17514438

[B37] Kandola A. Lewis G. Osborn D. P. J. Stubbs B. Hayes J. F. (2020). Depressive symptoms and objectively measured physical activity and sedentary behaviour throughout adolescence: a prospective cohort study. Lancet Psychiatry 7, 262–271. 10.1016/S2215-0366(20)30034-1 32059797 PMC7033559

[B38] Kearney M. W. (2017). Cross-lagged panel analysis, in The Sage Encyclopedia of Communication Research Methods, ed. Allen M. (California: SAGE Publications).

[B39] Khalife N. Kantomaa M. Glover V. Tammelin T. Laitinen J. Ebeling H.. (2014). Childhood attention-deficit/hyperactivity disorder symptoms are risk factors for obesity and physical inactivity in adolescence. J. Am. Acad. Child Adolesc. Psychiatry 53, 425–436. 10.1016/j.jaac.2014.01.009 24655652

[B40] Khan A. Burton N. W. (2021). Electronic games, television, and psychological wellbeing of adolescents: mediating role of sleep and physical activity. Int. J. Environ. Res. Public Health 18, 8877. 10.3390/ijerph18168877 34444625 PMC8393885

[B41] Klasen H. Woerner W. Wolke D. Meyer R. Overmeyer S. Kaschnitz W.. (2000). Comparing the German versions of the Strengths and Difficulties Questionnaire (SDQ-Deu) and the child behavior checklist. Eur. Child Adolesc. Psychiatry 9, 271–276. 10.1007/s007870070030 11202102

[B42] Kleppang A. L. Hartz I. Thurston M. Hagquist C. (2019). Leisure-time physical activity among adolescents and subsequent use of antidepressant and hypnotic drugs: a prospective register linkage study. Eur. Child Adolesc. Psychiatry 28, 177–188. 10.1007/s00787-018-1160-x 29721753 PMC6510848

[B43] Klipker K. Baumgarten F. Göbel K. Lampert T. Hölling H. (2018). Mental health problems in children and adolescents in Germany. Results of the cross-sectional KiGGS Wave 2 study and trends. J. Health Monit. 3, 34–41. 10.17886/RKI-GBE-2018-084 35586801 PMC8848775

[B44] Koning M. Jong A. de Jong E. de Visscher T. L. S. Seidell J. C. Renders C. M. (2018). Agreement between parent and child report of physical activity, sedentary and dietary behaviours in 9-12-year-old children and associations with children's weight status. BMC Psychol 6, 14. 10.1186/s40359-018-0227-2 29631618 PMC5891979

[B45] Kurth B. M. (2018). Editorial: Neues von und über KiGGS. J. Health Monit. 3, 3–7. 10.17886/RKI-GBE-2018-003 PMC8874287 35586178

[B46] Kurth B. M. Kamtsiuris P. Hölling H. Schlaud M. Dölle R. Ellert U.. (2008). The challenge of comprehensively mapping children's health in a nation-wide health survey: design of the German KiGGS-Study. BMC Public Health 8, 196. 10.1186/1471-2458-8-196 18533019 PMC2442072

[B47] Lambez B. Harwood-Gross A. Golumbic E. Z. Rassovsky Y. (2020). Non-pharmacological interventions for cognitive difficulties in ADHD: a systematic review and meta-analysis. J. Psychiatr. Res. 120, 40–55. 10.1016/j.jpsychires.2019.10.007 31629998

[B48] Lampert T. Müters S. Stolzenberg H. Kroll L. E. (2014). Messung des sozioökonomischen Status in der KiGGS-Studie: Erste Folgebefragung (KiGGS Welle 1). Bundesgesundheitsblatt Gesundheitsforschung Gesundheitsschutz 57, 762–770. 10.1007/s00103-014-1974-8 24950825

[B49] Larun L. Nordheim L. V. Ekeland E. Hagen K. B. Heian F. (2006). Exercise in prevention and treatment of anxiety and depression among children and young people. Cochrane Database Syst. Rev. CD004691. 10.1002/14651858.CD004691.pub2 16856055 PMC12742371

[B50] Lawlor D. A. Hopker S. W. (2001). The effectiveness of exercise as an intervention in the management of depression: systematic review and meta-regression analysis of randomized controlled trials. BMJ. 322, 763–767. 10.1136/bmj.322.7289.763 11282860 PMC30551

[B51] Liang X. Li R. Wong S. H. S. Sum R. K. W. Sit C. H. P. (2021). The impact of exercise interventions concerning executive functions of children and adolescents with attention-deficit/hyperactive disorder: a systematic review and meta-analysis. Int. J. Behav. Nutr. Phys. Act. 18, 68. 10.1186/s12966-021-01135-6 34022908 PMC8141166

[B52] Liu Q. Zhou Y. Xie X. Xue Q. Zhu K. Wan Z.. (2021). The prevalence of behavioral problems among school-aged children in home quarantine during the COVID-19 pandemic in China. J. Affect. Disord. 279, 412–416. 10.1016/j.jad.2020.10.008 33099056 PMC7543949

[B53] Loewen O. K. Maximova K. Ekwaru J. P. Asbridge M. Ohinmaa A. Veugelers P. J. (2020). Adherence to life-style recommendations and attention-deficit/hyperactivity disorder: a population-based study of children aged 10 to 11 years. Psychosom. Med. 82, 305–315. 10.1097/PSY.0000000000000787 32251098

[B54] Loewen O. K. Maximova K. Ekwaru J. P. Faught E. L. Asbridge M. Ohinmaa A.. (2019). Lifestyle behavior and mental health in early adolescence. Pediatrics 143, e20183307. 10.1542/peds.2018-3307 31004047

[B55] Ma L. Hagquist C. Kleppang A. L. (2020). Leisure time physical activity and depressive symptoms among adolescents in Sweden. BMC Public Health 20, 997. 10.1186/s12889-020-09022-8 32586303 PMC7318414

[B56] Mauz E. Lange M. Houben R. Hoffmann R. Allen J. Gößwald A.. (2019). Cohort profile: KiGGS cohort longitudinal study on the health of children, adolescents and young adults in Germany. Int. J. Epidemiol. 49, 375–375. 10.1093/ije/dyz231 31794018 PMC7266535

[B57] Mercurio L. Y. Amanullah S. Gill N. Gjelsvik A. (2021). Children with ADHD engage in less physical activity. J. Atten. Disord. 25, 1187–1195. 10.1177/1087054719887789 31838947

[B58] Miklós M. Komáromy D. Futó J. Balázs J. (2020). Acute physical activity, executive function, and attention performance in children with attention-deficit hyperactivity disorder and typically developing children: an experimental study. Int. J. Environ. Res. Public Health 17, 4071. 10.3390/ijerph17114071 32517384 PMC7312258

[B59] Nejati V. Derakhshan Z. (2021). The effect of physical activity with and without cognitive demand on the improvement of executive functions and behavioral symptoms in children with ADHD. Expert Rev. Neurother. 21, 607–614. 10.1080/14737175.2021.1912600 33849353

[B60] Neudecker C. Mewes N. Reimers A. K. Woll A. (2019). Exercise interventions in children and adolescents with ADHD: a systematic review. J. Atten. Disord. 23, 307–324. 10.1177/1087054715584053 25964449

[B61] Neville R. D. Guo Y. Boreham C. A. Lakes K. D. (2021). Longitudinal association between participation in organized sport and psychosocial development in early childhood. J. Pediatr. 230, 152–160.e1. 10.1016/j.jpeds.2020.10.077 33157074

[B62] Ng Q. X. Ho C. Y. X. Chan H. W. Yong B. Z. J. Yeo W. S. (2017). Managing childhood and adolescent attention-deficit/hyperactivity disorder (ADHD) with exercise: a systematic review. Complement. Ther. Med. 34, 123–128. 10.1016/j.ctim.2017.08.018 28917364

[B63] Nigg C. R. Wunsch K. Nigg C. Niessner C. Jekauc D. Schmidt S. C. E.. (2021). Are physical activity, screen time, and mental health related during childhood, preadolescence, and adolescence? 11-year results from the German Motorik-Modul longitudinal study. Am. J. Epidemiol. 190, 220–229. 10.1093/aje/kwaa192 33524119

[B64] Pagani L. S. Harbec M. J. Fortin G. Barnett T. A. (2020). Childhood exercise as medicine: extracurricular sport diminishes subsequent ADHD symptoms. Prev. Med. 141, 106256. 10.1016/j.ypmed.2020.106256 33002520

[B65] Parker A. G. Markulev C. Rickwood D. J. Mackinnon A. Purcell R. Alvarez-Jimenez M.. (2019). Improving Mood with Physical ACTivity (IMPACT) trial: a cluster randomised controlled trial to determine the effectiveness of a brief physical activity behaviour change intervention on depressive symptoms in young people, compared with psychoeducation, in addition to routine clinical care within youth mental health services-a protocol study. BMJ Open 9, e034002. 10.1136/bmjopen-2019-034002 31662409 PMC6830686

[B66] Pascoe M. Bailey A. P. Craike M. Carter T. Patten R. Stepto N.. (2020). Physical activity and exercise in youth mental health promotion: a scoping review. BMJ Open Sport Exerc. Med. 6, e000677. 10.1136/bmjsem-2019-000677 32095272 PMC7010991

[B67] Peralta G. P. Forns J. García de la Hera M. González L. Guxens M. López-Vicente M.. (2018). Sleeping, TV, cognitively stimulating activities, physical activity, and ADHD symptom incidence in children: a prospective study. J. Dev. Behav. Pediatr. 39, 192–199. 10.1097/DBP.0000000000000539 29261536

[B68] Posner J. Polanczyk G. V. Sonuga-Barke E. (2020). Attention-deficit hyperactivity disorder. Lancet 395, 450–462. 10.1016/S0140-6736(19)33004-1 31982036 PMC7880081

[B69] Poulain T. Vogel M. Meigen C. Spielau U. Hiemisch A. Kiess W. (2020). Parent-child agreement in different domains of child behavior and health. PLoS ONE 15, e0231462. 10.1371/journal.pone.0231462 32271851 PMC7145111

[B70] Qin Z. Shi L. Xue Y. Lin H. Zhang J. Liang P.. (2021). Prevalence and risk factors associated with self-reported psychological distress among children and adolescents during the COVID-19 pandemic in China. JAMA Netw. Open 4, e2035487. 10.1001/jamanetworkopen,0.2020.35487 33496797 PMC7838937

[B71] R Core Team (2020). R: A Language and Environment for Statistical Computing: In R Foundation for Statistical Computing. Vienna, Austria: R Core Team.

[B72] Reichert F. F. Menezes A. M. B. Araújo C. L. Hallal P. C. (2010). Self-reporting versus parental reporting of physical activity in adolescents: the 11-year follow-up of the 1993 Pelotas (Brazil) birth cohort study. Cad. Saude Publica 26, 1921–1927. 10.1590/S0102-311X2010001000008 20963289

[B73] Rodriguez-Ayllon M. Cadenas-Sánchez C. Estévez-López F. Muñoz N. E. Mora-Gonzalez J. Migueles J. H.. (2019). Role of physical activity and sedentary behavior in the mental health of preschoolers, children and adolescents: a systematic review and meta-analysis. Sports Med. 49, 1383–1410. 10.1007/s40279-019-01099-5 30993594

[B74] Rommel A. S. Lichtenstein P. Rydell M. Kuja-Halkola R. Asherson P. Kuntsi J.. (2015). Is physical activity causally associated with symptoms of attention-deficit/hyperactivity disorder? J. Am. Acad. Child Adolesc. Psychiatry 54, 565–570. 10.1016/j.jaac.2015.04.011 26088661 PMC4984951

[B75] Ruhland S. Lange K. W. (2021). Effect of classroom-based physical activity interventions on attention and on-task behavior in schoolchildren: a systematic review. Sports Med. Health Sci. 3, 125–133. 10.1016/j.smhs.2021.08.003 35784522 PMC9219312

[B76] Schuch F. B. Stubbs B. Meyer J. Heissel A. Zech P. Vancampfort D.. (2019). Physical activity protects from incident anxiety: a meta-analysis of prospective cohort studies. Depress. Anxiety 36, 846–858. 10.1002/da.22915 31209958

[B77] Schuch F. B. Vancampfort D. Richards J. Rosenbaum S. Ward P. B. Stubbs B. (2016). Exercise as a treatment for depression: a meta-analysis adjusting for publication bias. J. Psychiatr. Res. 77, 42–51. 10.1016/j.jpsychires.2016.02.023 26978184

[B78] Seiffer B. Hautzinger M. Ulrich R. Wolf S. (2021). The efficacy of physical activity for children with attention deficit hyperactivity disorder: a meta-analysis of randomized controlled trials. J. Atten. Disord. 10870547211017982. 10.1177/10870547211017982 34041952 PMC8785285

[B79] Sithole F. Veugelers P. J. (2008). Parent and child reports of children's activity. Health Rep. 19, 19–24. 18847142

[B80] Smith S. D. Crowley M. J. Ferrey A. Ramsey K. Wexler B. E. Leckman J. F.. (2019). Effects of Integrated Brain, Body, and Social (IBBS) intervention on ERP measures of attentional control in children with ADHD. Psychiatry Res. 278, 248–257. 10.1016/j.psychres.2019.06.021 31233935 PMC6637759

[B81] Takacs Z. K. Kassai R. (2019). The efficacy of different interventions to foster children's executive function skills: a series of meta-analyses. Psychol. Bull. 145, 653–697. 10.1037/bul0000195 31033315

[B82] Tandon P. S. Zhou C. Johnson A. M. Gonzalez E. S. Kroshus E. (2021). Association of children's physical activity and screen time with mental health during the COVID-19 pandemic. JAMA Netw. Open 4, e2127892. 10.1001/jamanetworkopen.2021.27892 34596669 PMC8486978

[B83] Thapar A. Cooper M. Rutter M. (2017). Neurodevelopmental disorders. Lancet Psychiatry 4, 339–346. 10.1016/S2215-0366(16)30376-5 27979720

[B84] Thomas R. Sanders S. Doust J. Beller E. Glasziou P. (2015). Prevalence of attention-deficit/hyperactivity disorder: a systematic review and meta-analysis. Pediatrics 135, e994–1001. 10.1542/peds.2014-3482 25733754

[B85] van der Niet A. G. Smith J. Scherder E. J. A. Oosterlaan J. Hartman E. Visscher C. (2015). Associations between daily physical activity and executive functioning in primary school-aged children. J. Sci. Med. Sport 18, 673–677. 10.1016/j.jsams.2014.09.006 25262450

[B86] van Woudenberg T. J. Bevelander K. E. Burk W. J. Buijzen M. (2020). The reciprocal effects of physical activity and happiness in adolescents. Int. J. Behav. Nutr. Phys. Act. 17, 147. 10.21203/rs.3.rs-76099/v1 33213465 PMC7678192

[B87] Vysniauske R. Verburgh L. Oosterlaan J. Molendijk M. L. (2020). The effects of physical exercise on functional outcomes in the treatment of ADHD: a meta-analysis. J. Atten. Disord. 24, 644–654. 10.1177/1087054715627489 26861158

[B88] Wegner M. Amatriain-Fernández S. Kaulitzky A. Murillo-Rodriguez E. Machado S. Budde H. (2020). Systematic review of meta-analyses: exercise effects on depression in children and adolescents. Front. Psychiatry. 11, 81. 10.3389/fpsyt.2020.00081 32210847 PMC7068196

[B89] Welsch L. Alliott O. Kelly P. Fawkner S. Booth J. Niven A. (2021). The effect of physical activity interventions on executive functions in children with ADHD: a systematic review and meta-analysis. Ment. Health Phys. Act. 20, 100379. 10.1016/j.mhpa.2020.100379

[B90] Wilens T. E. Spencer T. J. (2010). Understanding attention-deficit/hyperactivity disorder from childhood to adulthood. Postgrad. Med. 122, 97–109. 10.3810/pgm.2010.09.2206 20861593 PMC3724232

[B91] Wiles N. J. Jones G. T. Haase A. M. Lawlor D. A. Macfarlane G. J. Lewis G. (2008). Physical activity and emotional problems amongst adolescents: a longitudinal study. Soc. Psychiatry Psychiatr. Epidemiol. 43, 765–772. 10.1007/s00127-008-0362-9 18438732

[B92] Wilson B. Barnett L. M. (2020). Physical activity interventions to improve the health of children and adolescents in out of home care – a systematic review of the literature. Child. Youth Serv. Rev. 110, 104765. 10.1016/j.childyouth.2020.104765

[B93] Wolf S. Seiffer B. Zeibig J. M. Welkerling J. Brokmeier L. Atrott B.. (2021). Is physical activity associated with less depression and anxiety during the COVID-19 pandemic? A rapid systematic review. Sports Med 51, 1771–1783. 10.1007/s40279-021-01468-z 33886101 PMC8060908

[B94] World Health Assembly (2012). Global burden of mental disorders and the need for a comprehensive, coordinated response from health and social sectors at the country level: report by the Secretariat. World Health Organisation, 65. Available online at: https://apps.who.int/iris/handle/10665/78898 (accessed December 10, 2019).

[B95] World Health Organization (2010). Global Recommendations on Physical Activity for Health. Available online at: https://www.who.int/publications/i/item/9789241599979 (accessed February 15, 2020).

[B96] World Health Organization (2021). Child and Adolescents Mental Health. World Health Organisation, 2021. Available online at: https://www.who.int/mental_health/maternal-child/child_adolescent/en/ (accessed November 18, 2021).

[B97] Wu X. Bastian K. Ohinmaa A. Veugelers P. (2018). Influence of physical activity, sedentary behavior, and diet quality in childhood on the incidence of internalizing and externalizing disorders during adolescence: a population-based cohort study. Ann. Epidemiol. 28, 86–94. 10.1016/j.annepidem.2017.12.002 29439784

[B98] Wu X. Veugelers P. J. Ohinmaa A. (2021). Health behavior, health-related quality of life, and mental health among Canadian children: a population-based cohort study. Front. Nutr. 8, 638259. 10.3389/fnut.2021.638259 33777992 PMC7991792

[B99] Wu X. Y. Han L. H. Zhang J. H. Luo S. Hu J. W. Sun K. (2017). The influence of physical activity, sedentary behavior on health-related quality of life among the general population of children and adolescents: a systematic review. PLoS ONE 12, e0187668. 10.1371/journal.pone.0187668 29121640 PMC5679623

[B100] Xiong S. Li X. Tao K. (2017). Effects of structured physical activity program on chinese young children's executive functions and perceived physical competence in a day care center. Biomed Res. Int. 2017, 5635070. 10.1155/2017/5635070 29238718 PMC5697411

[B101] Xue Y. Yang Y. Huang T. (2019). Effects of chronic exercise interventions on executive function among children and adolescents: a systematic review with meta-analysis. Br. J. Sports Med. 53, 1397–1404. 10.1136/bjsports-2018-099825 30737201

[B102] Yang W. Wong S. H. S. Sum R. K. W. Sit C. H. P. (2021). The association between physical activity and mental health in children with special educational needs: a systematic review. Prev. Med. Rep. 23, 101419. 10.1016/j.pmedr.2021.101419 34150477 PMC8193140

[B103] Zang Y. (2019). Impact of physical exercise on children with attention deficit hyperactivity disorders: evidence through a meta-analysis. Medicine 98, e17980. 10.1097/MD.0000000000017980 31725664 PMC6867774

[B104] Zhang X. Zhu W. Kang S. Qiu L. Lu Z. Sun Y. (2020). Association between physical activity and mood states of children and adolescents in social isolation during the COVID-19 epidemic. Int. J. Environ. Res. Public Health 17, 7666. 10.3390/ijerph17207666 33096659 PMC7589310

