# Supplementary material for: Physical activity and the development of general mental health problems or attention-deficit hyperactivity disorder (ADHD) symptoms in children and adolescents: A cross-lagged panel analysis of long-term follow-up epidemiological data
Source: Front Behav Neurosci. 2022 Sep 13;16:933139. doi: 10.3389/fnbeh.2022.933139 (PMC9513200; doi:10.3389/fnbeh.2022.933139)
Supplement: Supplementary file 1 [file Table_1.DOCX]

Table 1A. The correlation matrix between variables at three time-points.

Appendix A

|  | (1) | (2) | (3) | (4) | (5) | (6) | (7) | (8) | (9) | (10) | (11) | (12) |
| --- | --- | --- | --- | --- | --- | --- | --- | --- | --- | --- | --- | --- |
| **Baseline** |  |  |  |  |  |  |  |  |  |  |  |  |
| SDQ-Emotion (1) | 1 |  |  |  |  |  |  |  |  |  |  |  |
| SDQ-Behavioral problems (2) | **0.3**  **(**14491) | 1 |  |  |  |  |  |  |  |  |  |  |
| SDQ-Hyperactivity inattention (3) | **0.32**  (14487) | **0.48**  **(**14491) | 1 |  |  |  |  |  |  |  |  |  |
| SDQ-Peer problems (4) | **0.36**  (14484) | **0.3**  (14486) | **0.26**  (14486) | 1 |  |  |  |  |  |  |  |  |
| SDQ-Prosocial behavior (5) | **-0.1**  **(**14495) | **-0.39**  (14498) | **-0.27**  (14494) | **-0.24**  (14494) | 1 |  |  |  |  |  |  |  |
| SDQ-Total (6) | **0.7**  **(**14477) | **0.71**  (14477) | **0.78**  (14477) | **0.64**  (14477) | **-0.35**  (14476) | 1 |  |  |  |  |  |  |
| Physical activity (7) | **-0.09**  (13963) | -0.02+  (13970) | -0.00  (13965) | **-0.15**  (13962) | **0.05**  (13986) | **-0.09**  (13949) | 1 |  |  |  |  |  |
| Socioeconomic status (8) | **-0.13**  (14347) | **-0.16**  (14353) | **-0.22**  (14351) | **-0.17**  (14347) | **0.06**  (14375) | **-0.24**  (14330) | **0.12**  (16382) | 1 |  |  |  |  |
| Body-mass index (9) | 0.02+  (14416) | 0.02+  (14421) | **-0.06**  (14418) | **0.13**  (14416) | 0.00  (14448) | 0.03*  (14397) | **-0.12**  (16520) | **-0.08**  (17168) | 1 |  |  |  |
| **Wave 1** |  |  |  |  |  |  |  |  |  |  |  |  |
| Emotion (10) | **0.38**  (6465) | **0.17**  (6466) | **0.19**  (6465) | **0.19**  (6465) | **-0.05**  (6469) | **0.33**  (6462) | **-0.07**  (7969) | **-0.11**  (8395) | 0.01  (8367) | 1 |  |  |
| SDQ-Behavioral Problems (11) | **0.16**  (6464) | **0.43**  (6465) | **0.31**  (6464) | **0.2**  (6464) | **-0.22**  (6468) | **0.39**  (6461) | **-0.04**  (7970) | **-0.13**  (8396) | 0.03*  (8368) | **0.28**  (8440) | 1 |  |
| SDQ-Hyperactivity inattention (12) | **0.15**  (6465) | **0.33**  (6466) | **0.54**  (6465) | **0.19**  (6465) | **-0.18**  (6469) | **0.45**  (6462) | **-0.06**  (7968) | **-0.17**  (8395) | **-0.07**  (8366) | **0.28**  (8439) | **0.46**  (8439) | 1 |
| SDQ-Peer problems (13) | **0.2**  (6462) | **0.22**  (6463) | **0.23**  (6462) | **0.4**  (6462) | **-0.16**  (6466) | **0.36**  (6459) | **-0.11**  (7967) | **-0.14**  (8393) | **0.07**  (8365) | **0.34**  (8438) | **0.29**  (8439) | **0.25**  (8437) |
| SDQ-Prosocial Behavior (14) | **-0.09**  (6463) | **-0.23**  (6464) | **-0.17**  (6463) | **-0.17**  (6463) | **0.38**  (6467) | **-0.23**  (6460) | 0.02+  (7968) | 0.03*  (8394) | -0.01  (8366) | **-0.11**  (8439) | **-0.37**  (8439) | **-0.24**  (8438) |
| SDQ-Total (15) | **0.32**  (6462) | **0.4**  (6463) | **0.47**  (6462) | **0.33**  (6462) | **-0.21**  (6466) | **0.55**  (6459) | **-0.1**  (7964) | **-0.2**  (8391) | 0.00  (8362) | **0.69**  (8436) | **0.70**  (8436) | **0.76**  (8436) |
| Physical activity (16) | **-0.08**  (6183) | 0.01  (6183) | 0.03+  (6182) | **-0.07**  (6183) | -0.01  (6186) | -0.03+  (6180) | **0.04**  (7689) | **0.04**  (8106) | **-0.04**  (8080) | **-0.10**  (8096) | 0.02+  (8096) | **0.04**  (8094) |
| Body-mass index (17) | **0.07**  (5457) | **0.04**  (5458) | **0.08**  (5457) | **0.12**  (5457) | 0.04*  (5461) | **0.11**  (5454) | **0.07**  (6830) | **-0.14**  (7196) | **0.63**  (7166) | **0.05**  (7226) | **0.05**  (7227) | -0.03+  (7225) |
| **Wave 2** |  |  |  |  |  |  |  |  |  |  |  |  |
| SDQ-Emotion (18) | **0.23**  (2598) | **0.14**  (2598) | **0.12**  (2597) | **0.15**  (2598) | **-0.06**  (2601) | **0.23**  (2597) | **-0.08**  (4212) | **-0.12**  (4474) | 0.03+  (4453) | **0.42**  (3861) | **0.17**  (3861) | **0.17**  (3860) |
| SDQ-Behavioral problems (19) | **0.09**  (2601) | **0.28**  (2601) | **0.24**  (2600) | **0.14**  (2601) | **-0.18**  (2604) | **0.29**  (2600) | -0.05*  (4215) | **-0.11**  (4478) | 0.04*  (4456) | **0.13**  (3865) | **0.44**  (3865) | **0.33**  (3864) |
| SDQ-Hyperactivity inattention (20) | 0.04  (2602) | **0.25**  (2602) | **0.37**  (2601) | **0.14**  (2602) | **-0.18**  (2605) | **0.33**  (2601) | **-0.07**  (4214) | **-0.16**  (4477) | 0.03+  (4455) | **0.17**  (3864) | **0.31**  (3864) | **0.59**  (3863) |
| SDQ-Peer problems (21) | **0.1**  (2596) | **0.12**  (2596) | **0.17**  (2595) | **0.28**  (2596) | **-0.13**  (2599) | **0.25**  (2595) | **-0.07**  (4212) | **-0.11**  (4474) | **0.05**  (4451) | **0.19**  (3860) | **0.21**  (3860) | **0.21**  (3859) |
| SDQ-Prosocial behavior (22) | -0.05+  (2599) | **-0.16**  (2599) | **-0.14**  (2598) | **-0.14**  (2599) | **0.32**  (2602) | **-0.18**  (2598) | 0.04+  (4213) | 0.01  (4475) | 0.02  (4452) | **-0.05**  (3863) | **-0.26**  (3863) | **-0.17**  (3862) |
| SDQ-Total (23) | **0.16**  (2595) | **0.28**  (2595) | **0.33**  (2594) | **0.25**  (2595) | **-0.19**  (2598) | **0.39**  (2594) | **-0.09**  (4209) | **-0.18**  (4469) | **0.06**  (4447) | **0.33**  (3858) | **0.39**  (3858) | **0.48**  (3857) |
| Physical activity (24) | -0.04+  (2525) | 0.01  (2525) | -0.01  (2524) | -0.03  (2525) | -0.02  (2528) | -0.04  (2524) | 0.04*  (4076) | 0.03+  (4338) | 0.03  (4315) | **-0.07**  (3774) | 0.01  (3774) | 0.01  (3773) |
| Body-mass index (25) | 0.02  (4913) | **0.05**  (4915) | **0.07**  (4914) | **0.12**  (4916) | 0.01  (4920) | **0.09**  (4913) | -0.00  (6092) | **-0.15**  (6384) | **0.61**  (6384) | 0.04*  (4552) | **0.05**  (4551) | 0.02  (4551) |

Table 1A. continue

|  | (13) | (14) | (15) | (16) | (17) | (18) | (19) | (20) | (21) | (22) | (23) | (24) | (25) |
| --- | --- | --- | --- | --- | --- | --- | --- | --- | --- | --- | --- | --- | --- |
| **Wave 1** |  |  |  |  |  |  |  |  |  |  |  |  |  |
| SDQ-Peer problems (13) | 1 |  |  |  |  |  |  |  |  |  |  |  |  |
| SDQ-Prosocial Behavior (14) | **-0.23**  (8437) | 1 |  |  |  |  |  |  |  |  |  |  |  |
| SDQ-Total (15) | **0.63**  (8436) | **-0.33**  (8434) | 1 |  |  |  |  |  |  |  |  |  |  |
| Physical activity (16) | **-0.09**  (8093) | 0.01  (8094) | **-0.04**  (8091) | 1 |  |  |  |  |  |  |  |  |  |
| Body-max index (17) | **0.14**  (7225) | -0.03+  (7226) | **0.06**  (7222) | **-0.18**  (6948) | 1 |  |  |  |  |  |  |  |  |
| **Wave 2** |  |  |  |  |  |  |  |  |  |  |  |  |  |
| SDQ-Emotion (18) | **0.17**  (3861) | -0.04+  (3861) | **0.34**  (3860) | **-0.07**  (3800) | 0.04+  (3436) | 1 |  |  |  |  |  |  |  |
| SDQ-Behavioral problems (19) | **0.18**  (3865) | **-0.24**  (3865) | **0.39**  (3864) | 0.01  (3804) | 0.04+  (3439) | **0.28**  (4507) | 1 |  |  |  |  |  |  |
| SDQ-Hyperactivity inattention (20) | **0.17**  (3864) | **-0.15**  (3864) | **0.48**  (3863) | 0.05*****  (3803) | -0.03  (3439) | **0.29**  (4506) | **0.5**  (4509) | 1 |  |  |  |  |  |
| SDQ-Peer problems (21) | 0.45  (3860) | **-0.16**  (3860) | **0.36**  (3859) | **-0.1**  (3799) | **0.09**  (3434) | **0.37**  (4504) | **0.27**  (4506) | **0.24**  (4504) | 1 |  |  |  |  |
| SDQ-Prosocial behavior (22) | **-0.15**  (3863) | **0.45**  (3863) | **-0.22**  (3862) | 0.01  (3802) | 0.00  (3438) | **-0.1**  (4506) | **-0.44**  (4507) | **-0.27**  (4506) | **-0.24**  (4504) | 1 |  |  |  |
| SDQ-Total (23) | **0.34**  (3858) | **-0.2**  (3858) | **0.56**  (3857) | -0.03  (3797) | 0.04+  (3433) | **0.7**  (4502) | **0.7**  (4502) | **0.76**  (4502) | **0.65**  (4502) | **-0.36**  (4502) | 1 |  |  |
| Physical activity (24) | **-0.06**  (3774) | -0.02  (3774) | -0.04+  (3773) | **0.19**  (3715) | **-0.1**  (3359) | **-0.12**  (4315) | 0.01  (4319) | 0.05*****  (4318) | **-0.11**  (4314) | 0.00  (4315) | **-0.07**  (4309) | 1 |  |
| Body-mass index (25) | **0.13**  (4551) | 0.00  (4552) | **0.08**  (4550) | **-0.14**  (4458) | **0.74**  (3954) | 0.04+  (3519) | **0.06**  (3523) | -0.02  (3522) | **0.11**  (3518) | 0.01  (3519) | **0.06**  (3513) | **-0.11**  (3502) | 1 |

Bold numbers: *p*<.001, **p*<.01, +*p*<.05
